# Molecular and Quantitative Genetic Differentiation in *Sitobion avenae* Populations from Both Sides of the Qinling Mountains

**DOI:** 10.1371/journal.pone.0122343

**Published:** 2015-03-30

**Authors:** Xianliang Huang, Deguang Liu, Da Wang, Xiaoqin Shi, Jean-Christophe Simon

**Affiliations:** 1 State Key Laboratory of Crop Stress Biology for Arid Areas (Northwest A&F University), Yangling, Shaanxi Province, China; 2 Key Laboratory of Integrated Pest Management on Crops in Northwestern Loess Plateau, Ministry of Agriculture, Yangling, Shaanxi Province, China; 3 College of Plant Protection, Northwest A&F University, Yangling, Shaanxi Province, China; 4 Department of Foreign Languages, Northwest A&F University, Yangling, Shaanxi Province, China; 5 Institut National de la Recherche Agronomique (INRA), unité mixte de recherche (UMR) 1349, Institut de Génétique, Environnement et Protection des Plantes (IGEPP), Domaine de la Motte, Le Rheu, France; Oklahoma State University, UNITED STATES

## Abstract

Quantitative trait differences are often assumed to be correlated with molecular variation, but the relationship is not certain, and empirical evidence is still scarce. To address this issue, we sampled six populations of the cereal aphid *Sitobion avenae* from areas north and south of the Qinling Mountains, and characterized their molecular variation at seven microsatellite loci and quantitative variation at nine life-history traits. Our results demonstrated that southern populations had slightly longer developmental times of nymphs but much higher lifetime fecundity, compared to northern populations. Of the nine tested quantitative characters, eight differed significantly among populations within regions, as well as between northern and southern regions. Genetic differentiation in neutral markers was likely to have been caused by founder events and drift. Increased subdivision for quantitative characters was found in northern populations, but reduced in southern populations. This phenomenon was not found for molecular characters, suggesting the decoupling between molecular and quantitative variation. The pattern of relationships between F*_ST_* and Q*_ST_* indicated divergent selection and suggested that local adaptation play a role in the differentiation of life-history traits in tested *S*. *avenae* populations, particularly in those traits closely related to reproduction. The main role of natural selection over genetic drift was also supported by strong structural differences in G-matrices among *S*. *avenae* populations. However, cluster analyses did not result in two groups corresponding to northern and southern regions. Genetic differentiation between northern and southern populations in neutral markers was low, indicating considerable gene flow between them. The relationship between molecular and quantitative variation, as well as its implications for differentiation and evolution of *S*. *avenae* populations, was discussed.

## Introduction

Barriers to gene flow presented by mountain ranges can have significant consequences for ecology and evolution of various organisms, and even lead to the isolation of populations. The isolation of populations may in turn cause allopatric speciation in different environments where reproductive isolation between populations could occur gradually and incidentally as a result of mutation and drift, and indirect effects of local selections (causing local adaptations) [1–2]. The Qinling Mountains (with highest peak of 3, 767 m) in Shaanxi Province of China, which extend for nearly 2,500 kilometers in the east-west direction, provide a unique scenario to study such effects. The mountain ranges are located in the transition zone between two macroclimatic regimes (i.e., subtropical and warm-temperate zones) [3]. The regions south of the mountains have subtropical characteristics with wet summers and warm winters, while the regions north of them belong to warm-temperate and temperate zones with relatively dry summers and cold winters [4].

Significant geographic variation in life-history traits (e.g., developmental time and fecundity) can occur among populations of a species separated by environmental barriers like the Qinling Mountains [4]. Determining the mechanisms that cause such variation is one of the basic objectives of ecological and evolutionary studies. It has long been recognized that geographic variation in life-history traits of an organism can include both genetic and environmental components [5]. The genetically-determined components of life-history trait variation are a significant factor which drives evolution by supplying raw material for evolutionary changes. Genetic variation can be affected not only by gene flow, but by natural selection, genetic drift, colonization history, mutation, and their interactions [6–7]. Among these causes, natural selection is generally considered as the major force generating changes in the level of genetic variation within and among populations (especially in heterogeneous environments), and thus driving adaptation and evolutionary changes [7–9]. A population’s ability of responding to changing environments depends on additive genetic variation for quantitative traits that are ecologically relevant [10]. Due to technical and logistic difficulties in measuring such variation, many studies rely solely on molecular markers to evaluate adaptive genetic variation, however, the degree to which molecular variation reflects quantitative genetic variation between populations is still controversial [10].

The cosmopolitan aphid (*Sitobion avenae* (Fab.)), a serious pest on cereal crops [11], offers a good model organism to study how differentiation can evolve among populations of small organisms with assumed high dispersal ability for several reasons. *Sitobion avenae* is widely distributed in areas around the huge Qinling Mountains, which can be a significant barrier for its dispersal, since its occurrence on wild hosts is rare above 2, 000 m in the mountains (D. L., personal observation). This aphid reproduces by cyclical or obligate parthenogenesis, and can be maintained clonally for an indefinite period under controlled laboratory conditions. So quantitative traits of interest can be evaluated by using clonal individuals, and the partitioning of phenotypic variation into its genetic and environmental components can be achieved. Finally, microsatellite markers have been developed for this species, allowing for evaluation of its molecular variation and population structure. In our previous study [4], considerable divergence in life-history traits among *S*. *avenae* populations from north and south of the Qinling Mountains was identified, but it is still not clear about the relative importance of different evolutionary forces in shaping and maintaining genetic variation in those populations. We hypothesize that the separation of Qinling Mountains can cause increasing quantitative trait divergence and local adaptation of *S*. *avenae* populations in northern and southern Shaanxi Province, thus creating different population structuring patterns in those areas. To do so, we compared populations of *S*. *avenae* collected from areas north of the Qinling Mountains (hereafter referred to as northern populations) to those from areas south of the mountains (hereafter referred to as southern populations) using both *Q*_*ST*_ (an index of differentiation in quantitative traits [12]) and *F*_*ST*_ (an index of differentiation in neutral genetic markers [12]). The aims of the present study were: (a) to characterize patterns of genetic differentiation among *S*. *avenae* populations at molecular traits (evaluated using seven microsatellite markers) and at quantitative life-history traits (measured under common laboratory conditions), (b) to assess the relationship between molecular and quantitative trait variation, and (c) to evaluate the relative importance of selection and drift in shaping and maintaining genetic variation between northern and southern populations.

## Materials and Methods

### Aphid sampling and colony establishment

From April to May in 2013, individuals of *S*. *avenae* were sampled from six locations including three each in areas north and south of the Qinling Mountains. The aphid clones came from wheat fields in the north (collected at Fuping, 34°46'46"N, 109°01'56"E; Tongchuan, 35°07'05"N, 109° 06'58"E; and Luochuan, 35°46'26"N, 109°24'29"E) and south (collected at Jinshui, 33°16'10"N, 107°47'13"E; Longting, 33°12'41"N, 107°38'31"E; and Mianxian, 33°11'36"N, 106°56'55"E) of the Qinling Mountains (no particular permissions were needed for sample collecting activities at sites mentioned above, and no endangered or protected species were involved in the collecting activities). Aphid samples were collected from the field, and insect colonies were maintained in the lab as described in detail previously in [9]. Briefly, over 15 wingless adults of *S*. *avenae* were collected in each of the five or more fields randomly selected at each location. Winter wheat seeds (*Triticum aestivum* cv. Aikang 58) were planted, and aphid colonies were kept in rearing rooms. For eliminating environmentally induced effects, the *S*. *avenae* populations were reared under common laboratory conditions for three generations before bioassays. Even without genetic changes among populations, maternal effects produce adaptive plastic responses that can be mistaken as local adaptation, but after three generations of cultivation under common laboratory conditions such effects can become negligible [13].

### Life-history data collection

Plants used in life-history tests were grown, and aphid life-history data were collected as described in detail previously in [9]. Briefly, wheat seedlings of one- to two-leaf stage (one per plant) received aphids transferred from rearing plants. Each plant with an aphid individual was enclosed with a transparent plastic cylinder (6 cm in diameter, 15 cm in height), and maintained in environmental growth chambers at 20±1°C, a relative humidity of 65±2%, and a photoperiod of 16:8 (L:D). Thirty clones were randomly selected from aphid colonies of each location for use in the tests, and three to four replicates were conducted for each clone. Test aphid individuals were observed twice daily from birth until the onset of reproduction, and molting occurrences and mortality counts were recorded. After reproduction started, numbers of offspring and dead aphids were recorded daily, and their offspring were then removed until the death of all test aphids.

### Quantitative trait analysis

Developmental time of first to fourth nymphal instars, the total developmental time of nymphs, adult lifespan, reproductive time, post-reproductive time, and lifetime fecundity were calculated as described in [9]. The abovementioned quantitative traits were analyzed with nested analyses of variance (nested ANOVAs) (sources of variance: ‘region’, ‘location’ nested within ‘region’, and ‘clone’ nested within ‘location’) in SAS [14]. The variance explained by ‘clone’ (δ^2^_clone_) was determined. When overall variation in ANOVA was significant, separation of treatment means was carried out using Tukey tests at α < 0.05. Data were log-transformed when needed to meet the assumptions of normality and homoscedasticity (i.e., homogeneity of variance) in ANOVA.

Principal component analysis (PCA) was performed with all quantitative traits measured above after the raw data were log-transformed [14]. The purpose of PCA is to quantify the significance of the variables that explained the differences in the abovementioned quantitative traits, and identify similarity in group data structures [9]. The first two PCA components (PC1 and PC2) were plotted using the PROC GPLOT procedure. The factor weightings of each replicate for PC1 from the PCA were calculated, and they were used as a composite life-history factor (i.e., PC1) in subsequent analyses.

Our bioassays use clonal genotypes, and this design allows us to estimate the total variance of a particular quantitative trait (*V*_*P*_), which can be partitioned into among-clone genetic components *V*_***G***_ (i.e., the broad-sense genetic variance) and within-clone components *V*_*E*_ (i.e., environmental variance or residual variance) [9]. Broad-sense heritabilities were calculated as described previously in [9]. Genetic variance and covariance estimates for life-history traits were obtained with the restricted maximum likelihood (REML) method implemented in the software VCE 6.0.2 [15]. The genetic correlation between traits *x* and *y* was calculated from the genetic covariance estimate (cov[*x*, *y*]) and their additive variances as *r* = cov(*x*, *y*)/[(*v*_*x*_) × (*v*_*y*_)]^0.5^ The Flury hierarchical method was utilized to compare the resulting G matrices in the software CPCrand [16]. Based on maximum likelihood, this method can identify structural differences among G matrices by analyzing their eigenvectors and eigenvalues as described in [17]). Briefly, the method can test, in order, the models of unrelated structure, partial common principal components, common principal components, proportionality, and equality (see also in [17]). The statistical significance of genetic correlations and broad-sense heritabilities was assessed with likelihood-ratio tests (LRTs) (for more details, see also in [9, 17].

*Q*_*ST*_ values were evaluated using genetic variances and defined as *Q*_*ST*_ = *V*_*GB*_ /(*V*_*GB*_ + 2*V*_*GW*_), where *V*_*GB*_ is the genetic component of the variance between population means and *V*_*GW*_ is the average genetic variance within populations [18]. *Q*_*ST*_ was estimated between each pair of populations, as well as between northern and southern regions. Differentiation among populations within a region was evaluated similarly as *Q*_*SR*_. Pairwise population *Q*_*ST*_ values (a global index of differentiation) were also computed for the composite life-history trait, which was the first component obtained from the PCA performed on abovementioned quantitative traits. Following Chapuis *et al*. [7], *Q*_*ST*_ was considered different from corresponding *F*_*ST*_ if their respective 95% confidence intervals did not overlap. Another method of evaluating the variation in genetic variances for comparison among characters and populations is the coefficient of genetic variance (*CV*_*G*_ = *V*_*G*_^1/2^/*X*, where *X* is the mean phenotype) [19].

### Microsatellite data collection and analysis

Over 40 clones per population were randomly selected and genotyped at seven microsatellite loci (Sm10, Sm12, S17b, Sm17, S16b, Sa4∑ and S5L) following the procedures described in Simon et al. [20].

Using identified microsatellite loci, population differentiation was assessed by calculating pairwise *F*_*ST*_ (θ) [21] between populations and between both regions. Deviations from Hardy- Weinberg equilibrium and unbiased estimates of *F*_*IS*_ (f) [21] were estimated. Microsatellite diversity was assessed by calculating unbiased estimates of gene diversity (*H*_*E*_) [22] for each sample. Region-specific *F*_ST_ (hereafter referred to as *F*_SR_), *F*_IS_, *H*_*E*_, *Ho* (observed heterozygosity), and *R*_S_ (allele richness) were also estimated and their differences between regions were tested by performing 1000 permutations of alleles among individuals. The abovementioned *F*-statistics and related tests were conducted in FSTAT version 2.9.3 [23].

We also analyzed the microsatellite data with an individual based principal component analysis (PCA) using the program PCA-GEN version 1.2.1 [24]. This analysis uses allele frequencies to define new variables (components) that can characterize the neutral genetic variation among populations. PCA is a preferable method for visualizing microsatellite data in situations of potential gene flow because traditional bifurcating trees constructed from genetic distances can be difficult to interpret [25].

## Results

### Differences in quantitative traits

Northern populations showed significantly reduced developmental time for the first, second and third instar nymphs, but not for the fourth instar nymphs (Table 1). The total developmental time of nymphs for northern populations was also significantly lower than that for southern populations. Southern populations had much higher lifetime fecundity than northern populations. Post-reproductive time and adult lifespan of southern populations were much higher than those of northern populations, whereas the reproductive time of southern populations was only slightly higher than that of northern populations. Similarly, all the abovementioned quantitative traits showed significant variation among populations within a region except the developmental time of fourth instar nymphs. Of the nine quantitative characters, eight differed significantly among populations within regions, as well as between the two regions.

**Table 1:** Mean phenotypic values (SE) of all life-history traits for *Sitobion avenae* populations from both sides of Qinling Mountains (i.e., northern and southern regions).

**Traits**	**Northern regions**	**Southern regions**	**Difference between regions**		**Difference among populations nested in regions**	
			***F***	***P***	***F***	***P***
DT1	1.66 (0.02)	1.75 (0.04)	*F*_1,215_ = 5.26	0.023	*F*_4,215_ = 7.48	< 0.001
DT2	1.51 (0.02)	1.65 (0.03)	*F*_1,215_ = 30.09	< 0.001	*F*_4,215_ = 5.26	< 0.001
DT3	1.54 (0.02)	1.69 (0.03)	*F*_1,213_ = 25.60	< 0.001	*F*_4,213_ = 8.30	< 0.001
DT4	2.02 (0.02)	2.00 (0.03)	*F*_1,212_ = 0.20	0.658	*F*_4,212_ = 1.66	0.161
DT5	6.73 (0.03)	7.09 (0.06)	*F*_1,212_ = 53.77	< 0.001	*F*_4,212_ = 9.96	< 0.001
Lifetime fecundity	56.54 (0.87)	72.11 (1.57)	*F*_1,199_ = 216.05	< 0.001	*F*_4,199_ = 131.23	< 0.001
Post-reproductive time	8.11 (0.30)	15.20 (0.74)	*F*_1,199_ = 259.10	< 0.001	*F*_4,199_ = 66.77	< 0.001
Adult lifespan	28.67 (0.47)	37.75 (0.58)	*F*_1,199_ = 353.29	< 0.001	*F*_4,215_ = 268.05	< 0.001
Reproductive time	20.56 (0.30)	22.55 (0.67)	*F*_1,199_ = 20.86	< 0.001	*F*_4,199_ = 111.25	< 0.001

Note: DT1-DT4, the developmental time of 1^st^ to 4^th^ instar nymphs; DT5, the total developmental time of nymphs; all traits but lifetime fecundity measured in days; lifetime fecundity, the number of offspring produced in a female’s life.

### Quantitative genetic differentiation

Genetic difference between northern and southern populations was shown in Table 2. There were no significant differences between northern and southern populations in genetic variance (δ^2^_clone_). However, the coefficient of genetic variance (*CV*_*G*_) and mean broad sense heritability (*H* ^*2*^) for the populations in the northern region were both significantly higher than those in the southern region.

**Table 2:** Comparison of within-region molecular and quantitative genetic differentiation between northern and southern regions.

**Parameters**	**Northern region**	**Southern region**	***P* value**
δ^2^_clone_	18.9913	7.4387	0.056
*CV*_*G*_	0.1417	0.0741	**0.048**
*H* ^*2*^	0.4736	0.3579	**0.034**
*Ho*	0.744	0.630	0.602
*R*_*S*_	2.3	3.7	1.000
*F*_*IS*_	-0.679	-0.550	1.000

Note: The genetic variance (δ^2^_clone_), the coefficient of genetic variance (*CV*_*G*_) and the broad sense heritability (*H* ^*2*^) were measured for each region and over all quantitative traits; allelic richness (*R*_*S*_), observed heterozygosity (*Ho*), and inbreeding coefficient (*F*_*IS*_) were estimated for each region and over all loci, and significance tests were performed between northern and southern regions by randomization procedures using FSTAT software.

Significant genetic correlations were found between life-history traits for *S*. *avenae* populations from both regions (Table 3). Significantly negative correlation (i.e., trade-off) between DT1 and DT2 was found for southern populations, but not for northern populations. DT1, DT2 and DT3 were all shown to be significantly and negatively correlated with FEC, POS, SPA and RET for northern populations, whereas all the genetic correlations were non-significant for southern populations except that DT2 was significantly correlated with FEC and SPA. DT5 was significantly and negatively correlated with FEC, POS, SPA and RET for northern populations, whereas it was only significantly correlated with FEC among the characters for southern populations. The correlations of POS-FEC and POS-RET were significantly positive for northern populations, but they were significantly negative for southern populations. The correlations of FEC-SPA, FEC-RET and SPA-RET were all significantly positive for populations from both regions.

**Table 3:** Genetic correlations between life-history traits for northern (above the diagonal) and southern (below the diagonal) populations of *Sitobion avenae*.

	**DT1**	**DT2**	**DT3**	**DT4**	**DT5**	**FEC**	**POS**	**SPA**	**RET**
DT1		-0.143	0.076	0.011	0.489***	-0.388*	-0.163*	-0.279*	-0.282*
DT2	-0.168*		-0.214*	-0.016	0.377*	-0.300*	-0.310*	-0.327*	-0.219*
DT3	-0.043	-0.226*		0.042	0.506***	-0.460***	-0.202*	-0.364*	-0.350*
DT4	-0.124	0.299*	-0.334*		0.468***	-0.150	-0.020	0.030	0.062
DT5	0.478**	0.438**	0.317*	0.437**		-0.471***	-0.281*	-0.339*	-0.299*
FEC	-0.018	-0.371**	-0.038	-0.285*	-0.263*		0.619***	0.852***	0.857***
POS	-0.090	-0.135	0.045	-0.133	-0.117	-0.404***		0.848***	0.527***
SPA	0.021	-0.191*	0.027	-0.082	-0.072	0.253*	0.494***		0.875***
RET	0.068	-0.063	-0.086	0.017	0.035	0.675***	-0.670***	0.305*	

Note: DT1-DT4, the developmental time of 1^st^ to 4^th^ instar nymphs; DT5, the total developmental time of nymphs; FEC, lifetime fecundity; POS, post-reproductive time; SPA, adult lifespan; RET, reproductive time; statistical significance of genetic correlations evaluated using likelihood-ratio tests;

*, *P* < 0.05;

**, *P* < 0.01;

***, *P* < 0.001.

G-matrix comparisons by Flury’s method and jump-up approach (that is, at each step in the hierarchy, the hypothesis is tested against the hypothesis of unrelated structure) showed significant differences between paired matrices of life-history traits (Table 4). The CPC(1) model best explained the differences between matrices for populations Luochuan and Tongchuan from the northern region (LRT = 35.6, *P* < 0.01), and between those for Longting and Mianxian from the southern region (LRT = 75.0, *P* < 0.001), in other words, matrices shared only one of the nine possible principal components. However, unrelated structures were found for all other comparisons (LRT = 74.0–199.6, *P* < 0.001). So, G-matrices comparison showed significant structural differences among populations within regions, as well as between regions. The strong structural differences identified can be interpreted as a result of divergent selection among populations.

**Table 4:** Comparisons of G-matrices for quantitative traits of *Sitobion avenae* populations from north and south of the Qinling Mountains.

**Population source**	**G matrices**	**Flury hierarchy**		
		**LRT**	***P*-value**	**Verdict**
Northern region	Fuping vs. Luochuan	199.6	< 0.001	Unrelated
	Fuping vs. Tongchuan	110.9	< 0.001	Unrelated
	Luochuan vs. Tongchuan	35.6	< 0.01	CPC(1)
Southern region	Jinshui vs. Longting	90.3	< 0.001	Unrelated
	Jinshui vs. Mianxian	74.0	< 0.001	Unrelated
	Longting vs. Mianxian	75.0	< 0.001	CPC(1)
Between the two regions		88.2	< 0.001	Unrelated

Note: The verdict of the Flury hierarchy is the model shown to be the best in explaining the difference between paired matrices; the *P*-values are for the test of equality of two matrices; CPC(1), one of the nine possible components shared in common; unrelated, no relations between the matrices.

### Microsatellite differentiation

Microsatellite variation among populations (Table 5) was evident, with overall gene diversity for southern populations ranging from 0.285 in population Mianxian to 0.766 in population Jinshui. Overall gene diversity for single northern populations varied from 0.393 in population Luochuan to 0.538 in population Fuping. So, variation in gene diversity among populations within a particular region tended to be higher in the south than in the north. Levels of gene diversity tended to be lower in the north (0.466 ± 0.049) than in the south (0.530 ± 0.086), although the difference was not significant (*P* > 0.05). The differences in allelic richness (*R*_*S*_), observed heterozygosity (*Ho*), and inbreeding coefficient (*F*_*IS*_) between the two regions were not significant (all *P* > 0.05, Table 2).

**Table 5:** Gene diversity (SE) for each locus in each population.

**Population**	**Sm10**	**Sm12**	**S17b**	**Sm17**	**S16b**	**Sa4∑**	**S5L**	**All loci**
Fuping	0.500	0.500	0.607	0.619	0.567	0.476	0.500	0.538 (0.022)
Tongchuan	0.500	0.650	0.350	0.650	0.500	0.250	0.350	0.464 (0.058)
Luochuan	0.500	0.500	0.350	0.600	0.600	0.200	0.000	0.393 (0.085)
Jinshui	0.833	0.808	0.802	0.771	0.825	0.731	0.594	0.766 (0.032)
Longting	0.500	0.500	0.000	0.500	0.833	0.000	0.000	0.333 (0.126)
Mianxian	0.500	0.500	0.000	0.500	0.500	0.000	0.000	0.285 (0.101)
All populations	0.576	0.622	0.377	0.631	0.654	0.305	0.260	0.489 (0.064)
Northern populations	0.501	0.550	0.436	0.623	0.561	0.309	0.283	0.466 (0.049)
Southern populations	0.671	0.760	0.318	0.645	0.754	0.313	0.248	0.530 (0.086)

### *Q*_*ST*_ versus *F*_*ST*_

Pairwise *Q*_*ST*_ and *F*_*ST*_ values for all tested *S*. *avenae* populations were shown in Table 6. Pairwise *F*_ST_ values ranged from 0 for population Longting vs. population Mianxian to 0.1228 for population Jinshui vs. population Mianxian (Table 6). Molecular variation was found to be very low between populations Luochuan, Tongchuan, Longting and Mianxian, with all of the pairwise *F*_*ST*_ values smaller than 0.021. Substantial molecular variation was found between population Fuping and population Longting (*F*_*ST*_ = 0.1068) or Mianxian (*F*_*ST*_ = 0.1083). Substantial molecular variation was also found between population Jinshui and population Longting (*F*_*ST*_ = 0.1197) or Mianxian (*F*_*ST*_ = 0.1228).

**Table 6:** Pairwise *Q*_*ST*_ calculated from a composite life-history trait (below the diagonal) and *F*_*ST*_ (above the diagonal) estimates based on seven microsatellite loci for *Sitobion avenae* populations (the composite life-history trait obtained from PC1 of PCA analysis of all tested life-history traits; *, significant differences between *Q*_*ST*_ and corresponding *F*_*ST*_).

	**Fuping**	**Luochuan**	**Tongchuan**	**Jinshui**	**Longting**	**Mianxian**
Fuping		0.0879	0.0648	0.0419	0.1068	0.1083
Luochuan	0.6215*		0.0102	0.0978	0.0035	0.0036
Tongchuan	0.5554*	0.0094		0.0700	0.0206	0.0209
Jinshui	0.0808	0.5936*	0.5240*		0.1197	0.1228
Longting	0.0278	0.3181*	0.2624*	0.0612		0.0000
Mianxian	0.0102	0.4519*	0.3845*	0.0377	0.0591	

Pairwise *Q*_*ST*_ values varied from 0.0094 for population Luochuan vs. population Tongchuan to 0.6215 for population Fuping vs. population Luochuan. In terms of quantitative traits, population Fuping was significantly different from population Luochuan (*Q*_*ST*_ = 0.6215) or Tongchuan (*Q*_*ST*_ = 0.5554). Population Luochuan from north of the mountains differed substantially from all of the populations from south with pairwise *Q*_*ST*_ values ranging from 0.3181 to 0.5936, and similar substantial variation was found between population Tongchuan and any population from south (pairwise *Q*_*ST*_ varied from 0.2624 to 0.524). However, differentiation in quantitative traits was low between population Fuping of the northern region and any population of the southern region (pairwise *Q*_*ST*_ varied from 0.0102 to 0. 0808). Quantitative trait differentiation between populations from south of the mountains was also found to be low with *Q*_*ST*_ values varying from 0.0377 to 0.0612. Of all the pairwise comparisons, eight of them showed significantly higher *Q*_*ST*_ than corresponding *F*_*ST*_.

**Fig 1:**
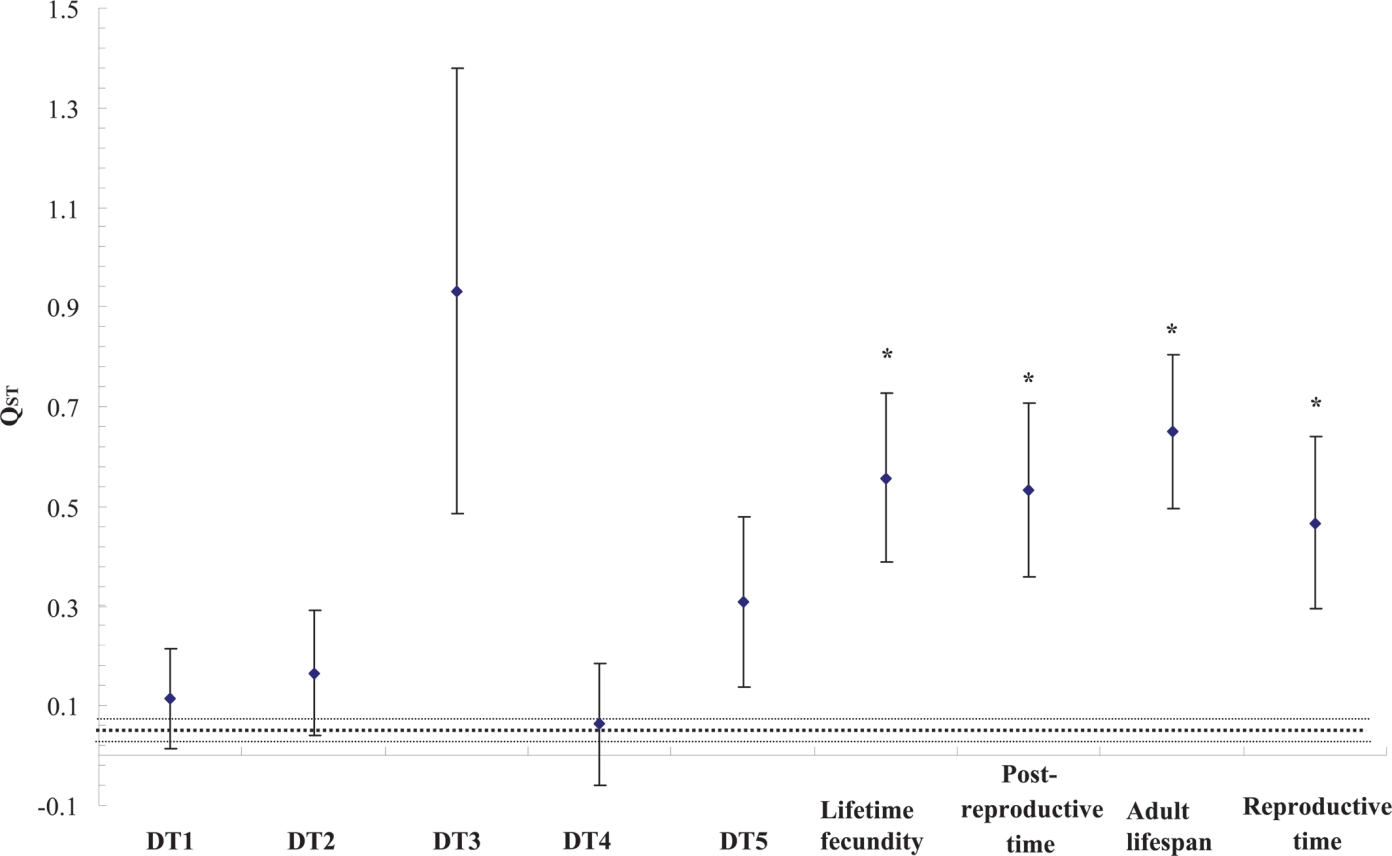
Differentiation (*Q*_*ST*_ ±SE) between northern and southern populations for different quantitative traits [the dotted line represents *F*_*ST*_ (±SE) between the two populations based on allelic variation in microsatellite loci; *, *Q*_*ST*_ significantly higher than *F*_*ST*_; DT1-DT4, the developmental time of 1^st^ to 4^th^ instar nymphs; DT5, the total developmental time of nymphs; *Q*_*ST*_, the index of quantitative variation based on life-history traits; *F*_*ST*_, the index of molecular variation derived from microsatellite markers].

The *Q*_*ST*_ values of the developmental times of first to fourth instar nymphs and the total developmental time of nymphs were not significantly higher than corresponding *F*_*ST*_ values (Fig. 1). However, lifetime fecundity, post-reproductive time, adult lifespan and reproductive time all showed significant differentiation among populations and significantly exceeded the neutral expectation set by *F*_*ST*_ (i.e., *Q*_*ST*_ > *F*_*ST*_).

**Fig 2:**
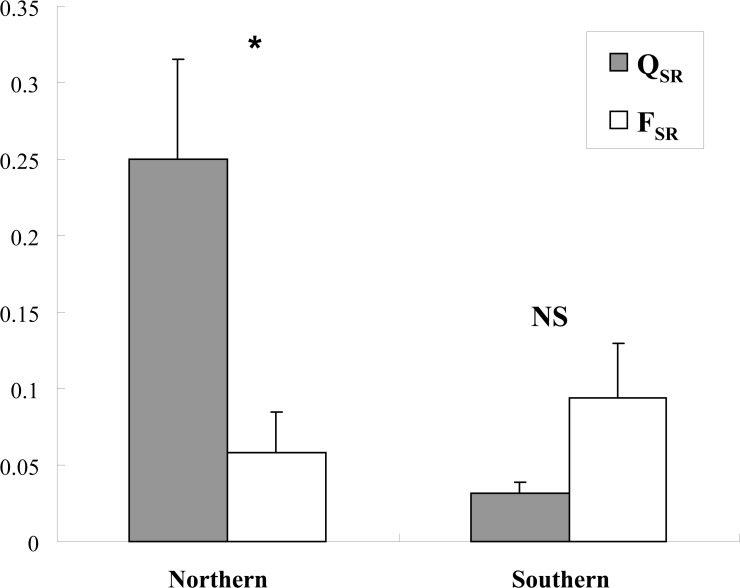
Comparison between molecular (*F*_*SR*_) and quantitative genetic (*Q*_*SR*_) divergence within each region (*, significant differences between *Q*_*SR*_ and *F*_*SR*_ based on non-overlapping confidence intervals; NS, non-significant differences between *Q*_*SR*_ and *F*_*SR*_; *F*_*SR*_ of the northern region was not significantly different from that of the southern region, *P* = 0.458).

The magnitude of subdivision in quantitative traits (i.e., *Q*_*SR*_) was significantly higher for northern populations than that for southern population, but there were no significant differences in molecular subdivision (i.e., *F*_*SR*_) between northern and southern regions (*P* > 0.05, Fig. 2). Quantitative trait differentiation among populations (*Q*_*SR*_) in the north was significantly higher than molecular subdivision among populations (*F*_*SR*_) in the same region. *F*_*SR*_ appeared to be higher than *Q*_*SR*_ in the south, but no significant differences were found between the two parameters.

### Principal component analyses (PCA)

**Fig 3:**
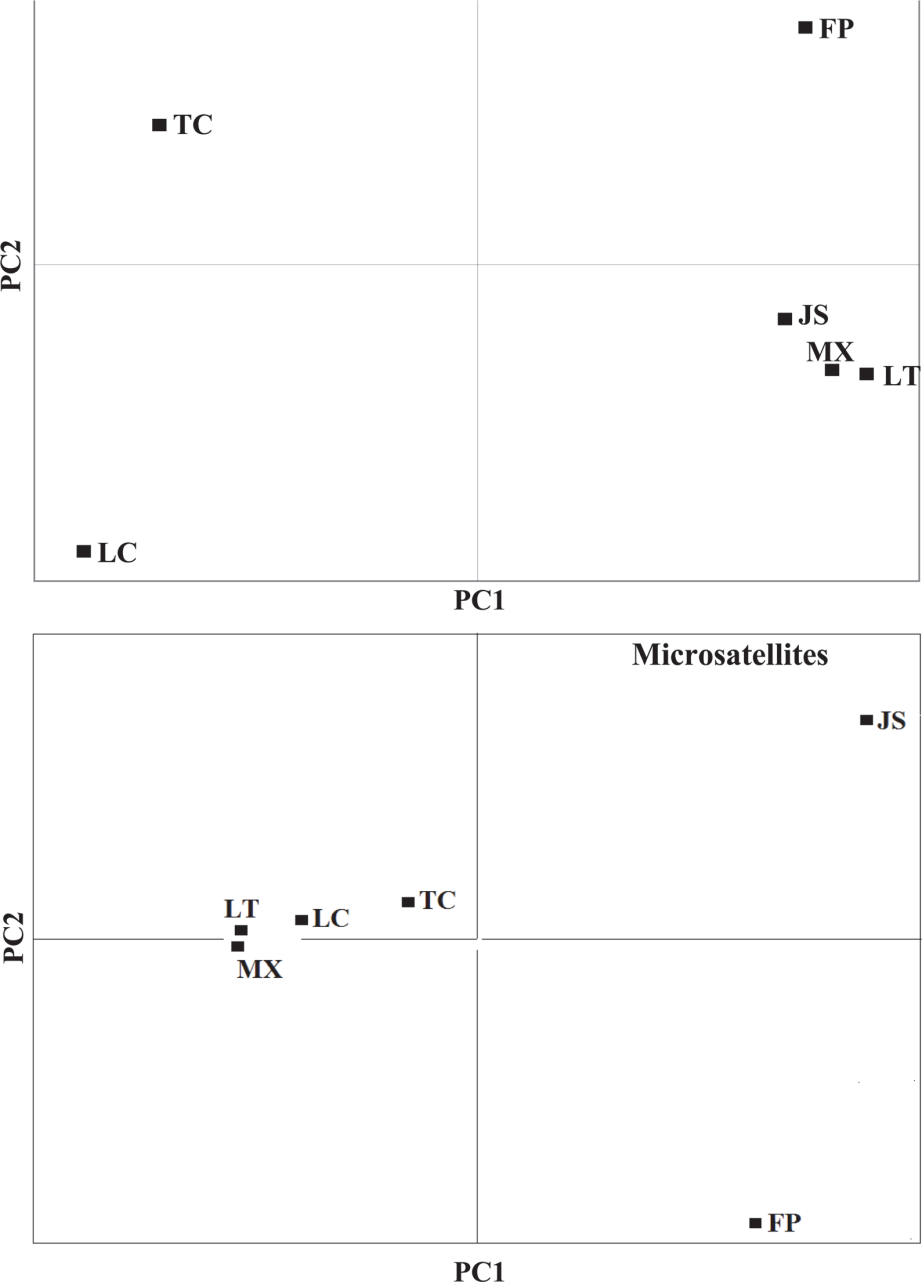
Plot of PC1 versus PC2 from principal component analysis of life-history traits (upper panel) and microsatellite loci (lower panel) as variables (northern populations: Fuping, FP; Luochuan, LC; Tongchuan, TC; southern populations: Jinshui, JS; Longting, LT; Mianxian, MX).

The first two components from PCA of life-history traits explained 91.4% of the total data variability (78.4% and 13.0% respectively for PC1 and PC2; Fig. 3, upper panel). Post-reproductive time (loading: 0.868) and adult lifespan (loading: 0.367) contributed the most to the first principal component (PC1). PC2 was associated mainly with reproductive time (loading: 0.596), lifetime fecundity (loading: 0.571), adult lifespan (loading: 0.316), and post reproductive time (loading: -0.451), the last one with negative correlation. The three southern populations (i.e., Jinshui, Longting and Mianxian) clustered together in the lower right of the plot, whereas the three northern populations (i.e., Fuping, Luochuan, and Tongchuan) were separated in the plot.

PCA on microsatellite data showed that the first two axes accounted for 95.6% of the total inertia (77.7% and 17.9% respectively for PC1 and PC2; Fig. 3, lower panel). This analysis clustered four populations (i.e., Longting, Mianxian, Luochuan and Tongchuan) together in the middle left of the plot, whereas the Jinshui and Fuping population fell in the upper and lower right of the plot, respectively.

## Discussion

### Relationship between molecular and quantitative variation

Many studies use molecular markers as a surrogate for adaptive genetic variation [10], and very few studies have addressed the relationship between molecular and quantitative subdivision for different populations of a species. *Sitobion avenae* provides a good model to explore this relationship because its clonal individuals can be reared in common laboratory conditions for several generations (to minimize confounding environmental effects) and then phenotyped [4], allowing for better estimates of heritable variation and comparisons between *Q*_*ST*_ and *F*_*ST*_.

In our study, northern and southern populations showed significantly different quantitative traits and G-matrices, so high quantitative variation was evident between populations from the two regions. However, microsatellite data did not show the expected variation between both types of populations. In addition, populations within the northern region had increased subdivision in quantitative traits (shown by higher *Q*_*SR*_ and *CV*_*G*_), but not in molecular characters. The decoupling of molecular and quantitative variation was also evident in PCA analyses where clustering patterns from molecular and quantitative trait data were distinct. The conversion of non-additive variation underlying quantitative traits to additive variation could occur due to isolation and drift following bottlenecks, and quantitative variation can be increased as a result [10, 26]. So, frequent bottlenecks may contribute to the decoupling of molecular and quantitative variation in our case, since *S*. *avenae* lives on highly seasonal and sometimes unpredictable habitats [27]. In our analyses of microsatellite markers, overall molecular differences between northern and southern populations appeared to be very low (*F*_*ST*_ = 0.018), indicating populations from the two regions shared the majority of identified genotypes. Therefore, high dispersal ability of *S*. *avenae* and the resulting gene flow can be an important factor in retarding the molecular differentiation between populations from both sides of the Qinling Mountains. The different pattern for molecular and quantitative traits might also be simply explained by differences in mutation rates, because mutation rates for quantitative characters are typically several orders of magnitude higher than those for molecular characters [28].

Indeed, the extent to which molecular markers reflect quantitative genetic subdivision between populations is still controversial [10]. Our results suggested that molecular variation might not be a reliable indicator for quantitative variation, and could only provide a conservative estimate of adaptive divergence. The decoupling of molecular and quantitative variation in our study is consistent with the findings of McKay and Latta [29], Palo et al. [30] and Chapuis et al. [7] that no significant correlations existed between molecular and quantitative variation, although positive correlations have been reported in both plants and animals [19, 31–32]. So, our results provided data cautioning against the use of molecular metrics of variation as a surrogate for the ability of *S*. *avenae* populations to respond to environmental changes. Further studies are still needed to clarify the relationship between molecular and quantitative variation in insects, as well as in other organisms.

### Differentiation and evolution of *S*. *avenae* populations

In our current study, all tested life-history characters but one (i.e., DT4) differed significantly between northern and southern regions, as well as among populations within a region. However, northern populations showed a higher DT4 than southern populations in our previous study [4]. Compared to those from northern populations, individuals from southern populations generally had longer developmental times, but higher fecundity, adult lifespan and reproductive time in this study. In contrast, no significant differences in DT2 and reproductive time were found between northern and southern populations in our previous study [4]. The seemingly contradictory results in some life-history traits may be attributed to different population sampling time of both studies since environmental conditions can vary significantly at the same location in the same month of different years. Despite the abovementioned inconsistency, consistent differences between northern and southern populations were found in DT1, post-reproductive time, adult lifespan, and lifetime fecundity in both studies. Northern and southern populations also differed significantly in genetic correlations between quantitative traits and in G-matrices. So, genetic variation among *S*. *avenae* populations from both sides of the Qinling Mountains was evident.

Between-population differentiation for neutral genetic markers—seven microsatellite loci—varied with *F*_*ST*_ ranging from 0 to 0.1228 (*F*_ST_ > 0.1 implies a very high degree of differentiation among populations, see more in Simon *et al*. [20] and Vialatte et al. [33]. The high genetic differentiation in neutral markers found between particular populations can be partially explained as a founder effect, due to fast growth and large population sizes after a bottleneck in population foundation by limited number of individuals. In several cases of pairwise comparisons (e.g., Luochuan vs. Tongchuan), no significant differences were found between *F*_*ST*_ and *Q*_*ST*_, so it can not be ruled out that genetic drift alone could account for the differentiation of *S*. *avenae* populations in those cases.

The role genetic drift plays in genetic differentiation of *S*. *avenae* populations is well documented [33–35], but the impact of natural selection has received little attention. *Sitobion avenae* showed a high potential to respond to natural selection, and rapid changes in ecologically relevant traits (e.g., phenotypic plasticity) have been reported in response to changes in selective pressure [27]. However, empirical data on the actual occurrence of local adaptation are scarce. In our study, differentiation of four quantitative traits closely related to reproduction (i.e., lifetime fecundity, post-reproductive time, adult lifespan and reproductive time) exceeded the neutral expectation set by *F*_*ST*_ (i.e., *Q*_*ST*_ > *F*_*ST*_), indicating divergent selection among *S*. *avenae* populations. There were over half pairwise population comparisons in the *Q*_*ST*_–*F*_*ST*_ analysis for the composite life-history trait that yielded higher *Q*_*ST*_ values than their corresponding *F*_*ST*_ estimates (i.e. more genetic divergence in the trait than expected by neutral genetic drift only), providing additional evidence of divergent selection. Populations of *S*. *avenae* that are better adapted to seasonal and ephemeral habitats are expected to have higher fitness under our experimental conditions (e.g. higher lifetime fecundity). In this study and [4], southern populations showed much higher fitness than northern populations, suggesting the occurrence of local adaptation. So, our results provide substantial evidence for patterns that suggest local adaptation in *S*. *avenae*. Another way to test for local adaptation is to perform reciprocal transfer of individuals in wild populations, and it is logistically demanding and extremely difficult to carry out such experiments [7,36]. However, such experiments may further substantiate the occurrence of local adaptation in *S*. *avenae*.

The occurrence of local adaptation in *S*. *avenae* is not unexpected in our study, because the separation of Qinling Mountains might cause the isolation of its populations. In addition, the pattern of natural selection can vary greatly between areas north and south to the Qinling Mountains because of different environmental conditions (esp., weather). The mountains cause harsh winter weather conditions in the north and mild ones in the south. The differences in winter weather conditions can have significant consequences for *S*. *avenae*’s life-history, for example, the reproductive mode of *S*. *avenae* clones in Romania (harsh winter) was different from that in France (mild winter) [34]. Thus, local adaptation, as a result of natural selection of local conditions, appears to be a common phenomenon for this insect. In this study and [4], natural selection seemed to favor individuals with smaller DT1, shorter post-reproductive time and shorter adult lifespan in the north of the mountains; on the contrary, those with larger DT1, longer post-reproductive time and longer adult lifespan were selected for in the south. We observed significant heritabilities, which show that populations have the potential to respond to local selection. As a result, it is likely that some individuals have adapted to local conditions.

Despite the highly likely occurrence of local adaptation in northern and southern populations, *S*. *avenae* individuals seemed to be able to disperse across the Qinling Mountains because cluster analyses using both quantitative trait (in this study and [4]) and microsatellite data didn’t result in two groups corresponding to northern and southern regions. The amount of dispersal of *S*. *avenae* individuals across the huge mountains was thought to be very low, because the Tianshan Mountains in Xinjiang of China led to clear geographic separation between northern and southern populations of a similar wheat aphid, *D*. *noxia* [37]. On the contrary, a substantial amount of *S*. *avenae* dispersal across the Qinling mountains could occur, because the overall low genetic differentiation between northern and southern populations (*F*_*ST*_ = 0.018) indicated a significant amount of gene flow between them. Therefore, the Qinling Mountains did not result in the isolation of *S*. *avenae* populations in our study area, and allopatric speciation for *S*. *avenae* appears to be highly unlikely.

*Sitobion avenae* presents a good model to study how differentiation can evolve among populations of small organisms with high dispersal ability, which is crucial to understand speciation and the match between environment and phenotype [12]. Further experiments are needed to determine what specific factors are important in differentiation and evolution of *S*. *avenae* populations. Temperature is a particularly likely candidate for the cause of differences between populations identified in our study. Because development is closely tied to temperature, natural selection might cause individuals from colder, northern climates to develop more rapidly than southern ones when raised under common laboratory conditions. The structure of G-matrix for *S*. *avenae*’s quantitative traits also has implications for its population differentiation and evolution. Interestingly, quite a few negative covariances (i.e., trade-offs) were found between quantitative traits measured in our study. These trade-offs (also shown by negative genetic correlations) may play a role in slowing the evolution of habitat specific *S*. *avenae* genotypes and thus slowing the differentiation of *S*. *avenae* populations in our study. The G-matrix structure of different quantitative traits for aphid genotypes have complex relationships with factors like genotype specialization, trade-offs, and genotype-by-environment interactions [38]. The identified strong structural differences in G-matrices for *S*. *aveane* populations represented a rare phenomenon, because empirical studies have generally supported the stability of G-matrices for populations of the same species [39]. Further studies are needed to explore the implications of G-matrix structure and stability in maintaining and shaping genetic differentiation in *S*. *avenae*, as well as in its evolutionary potential.
